# 肺癌骨转移诊疗专家共识（2019版）

**DOI:** 10.3779/j.issn.1009-3419.2019.04.01

**Published:** 2019-04-20

**Authors:** 北京医学奖励基金会肺癌青年专家委员会, 中国胸外科肺癌联盟

## 1 概述

我国肿瘤发病率以及死亡率在逐年升高，自2010年起，肿瘤已经成为首要死亡原因^[1-3]^。最新2018年国家癌症中心发布的数据显示，2014年肺癌发病率为57.13/10万人，总体发病人数78.2万，死亡人数为62.6万人，肺癌发病率以及死亡率仍居全国众癌之首^[4]^。且其发病隐匿，确诊时约50%为晚期（Ⅳ期），骨转移是主要的血行转移部位之一^[5]^。随着治疗方法和技术的进步，晚期肺癌患者的5年生存率逐渐提高^[6]^。患者生存获益的同时，发生骨转移及骨相关事件（skeletal related events, SREs）的风险亦随之增高^[7-10]^。

骨转移常预示患者生活质量的下降和生存期的缩短。引起的SREs，如骨痛、病理性骨折、脊髓压迫、高钙血症及相关治疗带来的痛苦等，严重影响患者的生活质量。在控制原发疾病的同时，积极预防和治疗骨转移骨相关事件尤为重要。在原发病的系统治疗基础之上，针对骨转移采取多学科综合治疗（mulitiple department treatment, MDT）模式，有计划、合理地制定个体化综合治疗方案，减少或延缓骨转移并发症及骨相关事件的发生，将有助于提高患者的生活质量。与以往恶性肿瘤骨转移诊疗共识不同，本共识特别增加了肺癌骨转移的心理治疗，强调肿瘤患者的身-心并重的整体治疗，符合生物-心理-社会医学模式，使患者最大程度获益。

## 2 发病率

肺癌骨转移发生率大约为10%-15%^[11]^，研究^[12]^显示甚至有50%的肺癌患者死后尸解发现有骨转移。肺癌骨转移后患者的中位生存时间仅为6个月-10个月，经过治疗后1年生存率也仅为40%-50%。肺癌骨转移的好发部位在脊柱和躯干骨近端。发生于脊柱者占50%，股骨占25%，肋骨和胸骨占12%^[7, 13, 14]^。

46%的肺癌骨转移患者并发SREs^[15]^。肺癌骨转移患者一旦发生SREs，将显著缩短患者生存期，有研究显示生存时间可缩短一半^[11]^。若合并严重SREs，如高钙血症、病理性骨折、脊髓压迫等并发症，患者的生存将进一步缩短^[11, 12]^。

## 3 病理与发病机制

恶性肿瘤骨转移按病变特征可分为以下三种类型：溶骨型、成骨型和混合型^[12, 16]^。成骨型骨转移常见于前列腺癌和膀胱癌，约占骨转移的10%。溶骨型骨转移占70%，常见于肺癌和乳腺癌^[12, 13, 16]^。骨转移致SREs是影响骨转移患者生活质量和生存期的直接因素。SREs发生危险性与恶性肿瘤类型相关。溶骨型病变为主的骨转移患者发生SRE危险性高。

肺癌骨转移主要是破骨细胞导致的骨吸收，大多表现为溶骨型病变^[17]^。肺癌细胞转移到骨后释放出可溶性介质，激活破骨细胞和成骨细胞。破骨细胞释放的细胞因子又进一步促进肿瘤细胞分泌骨溶解的介质，从而形成了恶性循环。应用抑制破骨细胞活性的药物，如双膦酸盐等可显著降低恶性骨转移瘤病灶内的破骨活动，降低由此引起的高钙血症和高尿钙症^[18]^

## 4 临床表现

仅50%肺癌骨转移患者出现临床症状^[12]^。肺癌骨转移常伴有严重骨痛及SREs（病理性骨折、脊髓压迫、高钙血症等）^[19, 20]^，不仅明显影响患者睡眠、情绪、日常生活能力，而且威胁患者的生存。骨痛为骨转移最主要的临床症状。随着肿瘤增大至骨髓腔内压力 > 6.67 kPa出现骨痛，且随病情进展逐渐加重。肿瘤分泌的前列腺素、白介素-1（interleukin-2, IL-2）、肿瘤坏死因子（tumor necrosis factor, TNF）等疼痛介质及肿瘤侵犯骨膜、神经、软组织均可导致剧烈疼痛（疼痛程度评估见附件1）。

病理性骨折常为肺癌骨转移癌的首发症状。约1/3患者以骨转移癌为首发症状而无原发癌表现^[21]^。在此前，患者可全无自觉症状，甚至带瘤生存数月至数年。高钙血症是肺癌骨转移的致死原因之一。肺癌骨转移晚期还可出现乏力、消瘦、贫血、低热。肺癌患者相关心理痛苦主要表现为焦虑、抑郁、失望及孤独等。因此，患者心理需求是大量的，如安全感、爱与被爱、理解、自尊等。如果这些需求得不到确认和较好的满足，就不可能获得疼痛及其他症状的缓解。

## 5 诊断

### 5.1 高危因素

原发肺癌病史的患者出现以下任何情况均可视为骨转移的高危人群，需进行骨转移相关检查：①骨痛/骨折；②脊髓或神经受压症状；③碱性磷酸酶升高；④高钙血症^[22]^。

### 5.2 诊断方法

对怀疑有骨转移的肺癌患者推荐其进行以下检查：

对怀疑有骨转移的肺癌患者推荐进行以下检查：

1. 放射性核素骨扫描(emission computed tomography, ECT)检查或正电子发射型计算机断层显像(positronemission tomography-computed tomography, PET-CT)检查

2. ECT检查阳性的部位行X线平片

3. ECT检查阳性的部位行CT及/或磁共振成像(magnetic resonance imaging, MRI)检查

#### 5.2.1 放射性核素显像

ECT与PET/CT是筛查骨转移的主要手段。目前ECT是骨转移首选的筛查方法^[23, 24]^，能够早期发现发生在骨骼中的成骨、溶骨或混合性骨转移灶，特别是对成骨性转移具有独特的优势。具有灵敏度高、全身骨组织一次成像不易漏诊的优点；但除了骨转移瘤之外的其他骨病变也可以出现核素浓聚，呈现出假阳性，因此ECT诊断骨转移的特异度较低^[25]^。PET/CT对于骨转移的灵敏度、特异度更高^[26]^，^18^F-FDG PET/CT对于溶骨及骨髓的转移最为敏感，而^18^F-NaF PET/CT对于成骨性转移最为敏感，因此选择恰当的显像剂对于骨转移寡病灶的诊断更为重要。^18^F-FDG PET/CT不仅可以反映全身骨骼受累的情况，同时还可以评价肿瘤的全身分期情况，其缺点是价格相对昂贵^[27]^。新型融合型显像设备PET/MR集成了PET及多参数MRI的多重优势，可能会较PET/CT发现更多、更小或更早的骨转移病灶，但价格昂贵、临床普及性差，临床应用效价比有待进一步观察^[28]^。

#### 5.2.2 X线

X线是常规的骨科检查方法，但X线平片检测早期骨转移瘤的灵敏度低^[7, 29]^，难以发现早期转移灶。常比ECT显示骨转移灶晚3个月-6个月，骨髓内转移未累积皮质时，易被高密度皮质掩盖而漏诊。故X线并不作为骨转移的常规检查手段，而是常用于对有临床症状的部位（如：疼痛、病理骨折）或其他影像学检查（如：ECT或MRI）所发现的异常进行补充评估^[30]^。X线平片有一定的特异性，其操作简单、费用低廉，仍是诊断骨转移的主要诊断工具^[22]^。

#### 5.2.3 CT/增强CT

CT较常规X线平片检测骨转移瘤的灵敏度高，是对骨转移的诊断、骨质破坏程度评价较实用的工具。它可更精确地显示骨质破坏及其周围软组织肿块；增强CT有助于显示骨转移瘤的血供特点、病变与周围神经、血管结构的关系。并且有助于判断脊柱的转移瘤组织是否突入椎管、压迫硬膜囊及神经根。CT对全身骨显像检查阳性而X线平片阴性、有局部症状、疑有骨转移、MRI禁忌的患者较有价值。而对于骨皮质的早期转移、骨转移骨髓质的浸润，CT诊断的敏感性较低。

#### 5.2.4 MRI检查

MRI对于骨转移的诊断有较高的敏感性和特异性，能通过多平面、多序列成像观察，更准确地显示转移侵犯部位、范围及周围软组织侵犯情况；增强MRI有助于显示更多转移灶。MRI有优于全身骨显像的敏感性，可显示ECT无法显示的早期骨转移灶，尤其适用于检测脊柱的转移灶，伴有神经症状的患者。当怀疑骨转移，全身骨显像和X线平片仍不能确定时，可行MRI检查提供诊断证据。MRI对骨髓腔内的早期转移灶有很高的灵敏度，是评价骨转移骨髓内浸润的首选工具。且MRI有助于骨转移与其他骨病变的鉴别，如感染性病变、良恶性骨折等。但MRI对于四肢长骨，尤其是皮质骨转移的作用有一定限度。近年来有研究显示，全身MR扫描技术可弥补常规MR扫描范围局限的问题，其诊断骨转移的敏感性同PET-CT ^[31]^；PET/MRI也显示出了潜在价值^[32]^；并且，在骨转移的治疗监测方面，影像学工具包括MRI也有一定的实践意义^[33]^；但这些工作尚未在临床实践中全面开展。

#### 5.2.5 骨活组织检查

病理学诊断是肺癌骨转移确诊的金标准。临床实践原则为：如果肺癌诊断明确，且全身多发骨破坏病灶，骨活检为非必须操作；如果肺癌诊断明确，但仅出现孤立性骨破坏病灶，则应积极进行活检。骨转移病灶的活检应遵循肌肉骨骼系统肿瘤活检取材的原则，多在CT引导或超声引导下进行，采用穿刺针切割或抽取肿瘤组织，慎用外科切开活检。穿刺活检前应尽量行增强CT或MR扫描，避开坏死区域取材且尽可能选取溶骨性区域取材，以满足常规病理及分子病理学诊断的要求。通常情况下，穿刺活检不会引起病理性骨折事件的发生。

#### 5.2.6 骨代谢的生物化学标记（bone biomarkers）

可反映骨转移过程中骨吸收和形成的速度，提示骨破坏和修复程度，是近期发现可潜在用于诊断及监控疾病进展的新技术，但因目前尚无前瞻性研究，除碱性磷酸酶（alkaline phosphates, ALP）外，暂不建议临床常规使用。①反映溶骨代谢水平的标记：Ⅰ型胶原羧基末端肽（carboxyterminal propeptide of type Ⅰ procollagen, ICTP）、Ⅰ型胶原N末端肽（N-telopeptide of type Ⅰ collagen, NTX）、Ⅰ型胶原α1羧基末端肽（CTX）、骨唾液蛋白（bone sialoprotein, BSP）等；②反映成骨代谢水平的标记：骨特异性碱性磷酸酶（bone alkaline phosphatase, BALP）、ALP、Ⅰ型溶胶原N末端肽（procollagen Ⅰ N-terminal propeptide, PINP）等^[34, 35]^。

### 5.3 诊断标准

肺癌骨转移的诊断应满足以下两个条件之一：①临床或病理诊断肺癌，骨病变活检符合肺癌转移；②肺癌病理诊断明确，具有典型的骨转移影像学表现。

### 5.4 诊断流程

见图 1。

**1:**
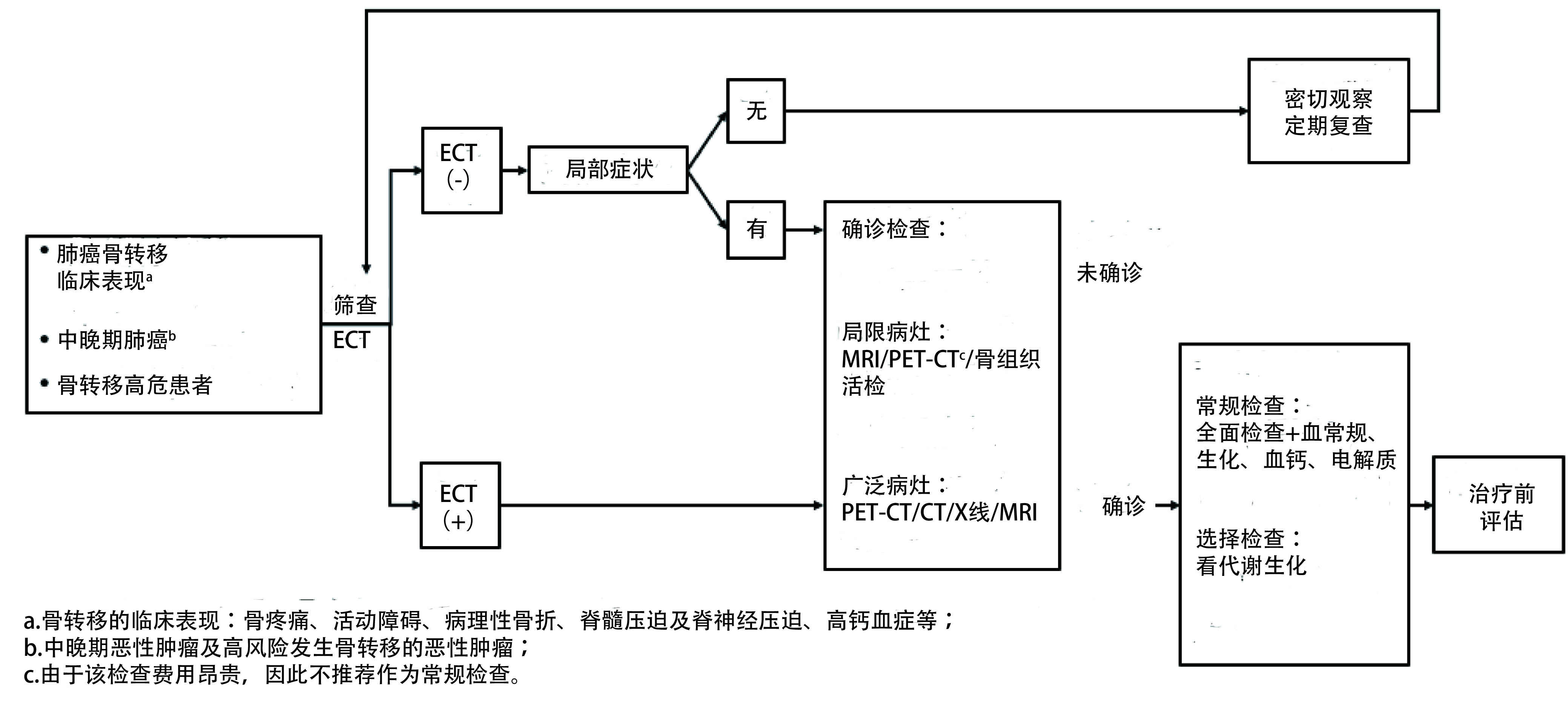
诊断流程

## 6 治疗

治疗肺癌骨转移的目标是提高生活质量、延长生命、缓解症状及心理痛苦、预防或延缓病理性骨折等SREs。肺癌出现骨转移时即为全身性疾病，应采取以全身治疗为主的综合治疗方式，包括：肺癌（原发病）的系统治疗（化疗、分子靶向治疗或免疫治疗）、放疗、手术、止痛、双膦酸盐和心理支持治疗。

治疗原则：以全身治疗为主，其中化疗、分子靶向治疗、免疫治疗可作为肺癌的抗肿瘤治疗方式，具体原则可参照原发性肺癌诊疗指南（2018版）。合理的局部治疗可以更好地控制骨转移相关症状，其中手术推荐用于治疗孤立骨转移灶，放射治疗也是有效的局部治疗手段。双膦酸盐可以预防和延缓SRE的发生。对症止痛治疗可明显改善患者的生活质量。应根据患者的机体状况、肿瘤病理学类型、病变累及范围（临床分期）和发展趋势，采取多学科综合治疗（multi-disciplinary therapy, MDT）模式，有计划、合理地制定个体化综合治疗方案。

主要抗肿瘤方法适应证：

化疗、分子靶向治疗、免疫治疗：多发骨转移灶

镇痛治疗：骨痛

骨改良药：诊断明确的骨转移

放疗：孤立骨转移灶（体外放射治疗），多发骨转移灶（放射性核素治疗），脊髓外压迫，外周神经肿瘤性压迫或侵犯所致疼痛或功能障碍

手术：病理性骨折及脊髓压迫固定术

介入治疗：消融治疗、骨成形术、近距离治疗

心理治疗：出现抑郁或焦虑的患者

### 6.1 骨改良药物

推荐双膦酸盐和地诺单抗（denosumab, D-mab）用于肺癌骨转移的治疗，无论是否有相应症状，在预防SREs发生方面，患者均可从治疗中受益。

#### 6.1.1 双膦酸盐

双膦酸盐是肺癌骨转移的基础用药，可以和常规抗肿瘤治疗（化疗、靶向治疗、放疗、放射性核素治疗和手术治疗）联合使用。双膦酸盐是焦膦酸盐分子的稳定类似物，以P-C-P基团取代焦磷酸盐结构中的P-O-P基团，改变焦磷酸盐的理化性质，增加其对水解酶的稳定性，改变其生物学性质及毒理作用。其中一条侧链使钙离子晶体与骨矿化基质（羟磷灰石）高度亲和，另一条侧链的差别使不同的双膦酸盐抗骨吸收的能力不同。新一代双膦酸盐（如伊班膦酸）较第一代双膦酸盐羟乙膦酸的体外作用强1, 000倍-100, 000倍^[36]^。研究表明双膦酸盐治疗骨转移的机制包括：①可以被破骨细胞选择性吸收，并选择性抑制破骨细胞活性，诱导破骨细胞凋亡，从而抑制骨吸收；②抑制破骨细胞成熟；③抑制成熟破骨细胞的功能；④抑制破骨细胞在骨质吸收部位的聚集；⑤抑制肿瘤细胞扩散、浸润和粘附于骨质。双膦酸盐能抑制破骨细胞对骨小梁的溶解和破坏，因此能阻止肿瘤转移引起的溶骨型病变、减少骨吸收、减轻骨痛及由骨转移所致的高钙血症及其他SREs^[37, 38]^。另外，已有多项研究^[36, 39-42]^显示，部分双膦酸盐有直接抗肿瘤作用，可抑制肿瘤细胞浸润和骨基质的粘附性，阻断肿瘤细胞释放破坏骨质释放的细胞因子和生长因子，并可诱导肿瘤细胞凋亡。

第一代双膦酸盐药物（氯膦酸等）和第二代双膦酸盐药物（帕米膦酸二钠、阿仑膦酸钠）能减轻肿瘤骨转移患者疼痛、预防或延缓SREs和提高患者生活质量。第三代双膦酸盐药物唑来膦酸、伊班膦酸钠和因卡膦酸二钠在此基础上，还能显著降低恶性肿瘤骨转移的高钙血症，增加骨质密度，减少骨代谢紊乱。对于骨转移伴严重疼痛的患者，伊班膦酸负荷剂量可快速缓解肿瘤骨转移患者的疼痛^[43]^。双膦酸盐治疗骨转移的剂量和给药方案见7.6双膦酸盐治疗推荐^[44]^。

双磷酸盐药物治疗骨转移的用法用量

1. 氯膦酸盐片剂1, 600 mg/d，口服给药；或氯膦酸盐针剂300 mg/d，静脉注射，>2 h持续5 d，之后换成口服给药；

2. 帕米膦酸盐90 mg，静脉注射>2 h，每3周-4周重复一次；

3. 唑来膦酸盐4 mg，静脉注射>15 min，每3周-4周重复一次；

4. 伊班膦酸盐6 mg，静脉注射>15 min，每3周-4周重复一次；伊班膦酸负荷疗法6 mg，静脉注射>15 min，连续3 d，持续每3周-4周重复一次；

5. 因卡膦酸二钠10 mg，65周岁以上患者推荐剂量为一次5 mg，静脉注射2 h-4 h，每3周-4周重复一次。

#### 6.1.2 地诺单抗（denosumab, D-mab）

地诺单抗是一种有独特作用机制的骨吸收抑制剂，其特异性靶向核因子κB受体活化因子配体（receptor activator of NF-κB ligand, RANKL），抑制破骨细胞活化和发展，减少骨吸收，增加骨密度。目前美国国立综合癌症网络（National Comprehensive Cancer Network, NCCN）指南及欧洲肿瘤内科学会（European Society for Medical Oncology, ESMO）指南中均推荐D-mab可用于肺癌骨转移的治疗，但是目前本药尚未在国内上市。

#### 6.1.3 适应证

肺癌患者影像学检查提示有骨破坏或骨转移时，如无应用禁忌证，均推荐应用骨改良药物治疗，只存在骨转移风险（LDH或ALP增高）但未确诊骨转移的患者不推荐使用骨改良药物治疗。

#### 6.1.4 用药时间及停药指征

一旦确诊肺癌骨转移应考虑给予骨改良药物治疗。研究证明双膦酸盐用于治疗骨转移的中位时间为9个月-18个月。因此，除非不能耐受该类药物的不良反应或出现禁忌证，推荐至少应持续用药9个月以上，并根据患者获益情况考虑是否长期用药。停药指征：用药过程中出现明确与骨改良药物治疗相关的严重不良反应；或临床医生认为继续用药患者不能获益。另外研究表明患者治疗期间出现骨痛加重或SREs时，继续接受唑来膦酸治疗，还可以降低再次发生SREs的风险，因此在应用双膦酸盐治疗过程中即使发生SREs仍建议继续用药，换药是否获益还有待更多的临床研究结果证实。临床上应根据患者安全性和获益情况采用个体化的用药时间。

#### 6.1.5 不良反应及用药注意事项

骨改良药物具有较好的耐受性，主要不良反应包括：颌骨坏死、肾毒性、低钙血症以及流感样症状（骨痛、发热、疲乏、寒战及关节或肌肉痛），此外偶有注射部位的轻度反应及不需治疗的无症状血浆磷酸盐水平降低等。少有因不良反应而中断治疗者，未见长期不良反应^[36]^。

##### 6.1.5.1 颌骨坏死（osteonecrosis of the jaw, ONJ）

双膦酸盐所导致的ONJ发生率相当，长期应用双膦酸盐会增加ONJ发生风险^[45, 46]^。拔牙、植牙、牙周疾病及口腔感染是ONJ的主要危险因素，科学预防可以有效降低发生ONJ的风险。因此在初始治疗前应该进行口腔检查及预防性治疗并且保持良好的口腔卫生状态。一旦开始静脉双膦酸盐治疗，应尽量避免侵入性口腔科操作，如果必须进行牙科手术时，应尽量保守处理，减少手术操作范围，一些研究者推荐在进行侵入性操作前的1个月-3个月，应该暂停使用静脉双膦酸盐，待口腔内伤口愈合后再继续双膦酸盐治疗，另外加用抗菌药物也有助于预防ONJ发生^[47]^。建议至少每6个月进行全面的口腔检查，一旦出现ONJ应早期采取积极治疗。

##### 6.1.5.2 肾毒性

双膦酸盐可以导致患者肾功能受损，但是大多数为1级-2级并且具有可逆性。发生肾功能受损的危险因素包括年龄 > 65岁、合并使用非甾体抗炎药（nonsteroidal antiinflammatory drugs, NSAIDs）接受含顺铂方案治疗以及患糖尿病或多发性骨髓瘤。一般中位发生时间为4.7个月-5.4个月^[48, 49]^，当血清肌酐清除率 < 60 mL/min发生急性肾功能不全的风险增加^[47, 48]^。对于轻中度肾功能不全患者（肌酐清除率≥30 mL/min），静脉输注唑来膦酸需要根据血清肌酐清除率进行减量，而伊班膦酸和帕米膦酸则无需调整剂量，建议对于轻中度肾功能不全患者帕米膦酸（90 mg），输注时间应大于4 h，对于严重肾功能不全患者（Ccr < 30 mL/min），伊班膦酸则需要减量至2 mg，每3周-4周1次，并且每次输注时间应超过1 h，不推荐静脉输注帕米膦酸和唑来膦酸。其他双膦酸盐应根据产品说明书进行减量或延长输注时间^[50-53]^。

##### 6.1.5.3 低钙血症

在应用双膦酸盐治疗期间未补充维生素D和口服钙剂可以使低钙血症的发生率升高5倍-6倍，如果治疗前血钙低于正常水平或肾功能不全均可进一步增加低钙血症发生的风险^[43, 45, 54, 55]^。因此在双膦酸盐治疗期间应注意每日补充适量维生素D和口服钙剂（500 mg/d），在治疗初期和治疗期间监测血钙变化。

鉴于可能存在上述风险，建议临床医生在使用双膦酸盐药物和D-mab之前应重点关注血肌酐、血清钙、磷酸盐和镁等指标，提前进行口腔检查；选择药物时应考虑患者的一般状况、疾病的总体情况、基础肾功能以及合并用药；用药期间密切监护患者健康状况，应针对患者不同状况调整治疗方案，最大程度地保障患者的用药安全。

### 6.2 药物治疗

#### 6.2.1 化疗

肺癌骨转移患者治疗前一般情况评估见附件2体能状态（performance status, PS）评分。化疗适应证：PS评分≤2分（附件3，ZPS评分，5分法），重要器官功能可耐受化疗。对于小细胞肺癌（small cell lung cancer, SCLC）的化疗PS评分可放宽至3分。

含铂双药联合化疗是晚期驱动基因阴性肺癌患者的标准一线方案，在缓解率和生存期方面均显著优于单药方案。推荐以顺铂或卡铂为基础的含铂双药方案。全身化疗可改善患者一般情况，提高生活质量。对于骨转移，一般需同时联合双膦酸盐药物，具体参考见《NCCN非小细胞肺癌临床实践指南2019.V1》中复发和转移一章、《ESMO转移性非小细胞肺癌临床实践指南》中骨转移治疗以及《原发性肺癌诊疗指南（2018版）》^[56-58]^，鼓励患者参加临床试验。

#### 6.2.2 分子靶向治疗

肺癌的分子靶向治疗是针对可能导致细胞癌变的驱动基因，从分子水平上阻断肿瘤信号传导通路，从而抑制肿瘤细胞生长，诱导凋亡，甚至使其完全消退的全新的生物治疗模式。根据药物的作用靶点，肺癌常用的分子靶向治疗药物包括：

（一）以表皮生长因子受体（epidermal growth factor receptor, *EGFR*）突变为靶点EGFR酪氨酸激酶抑制剂（EGFR tyrosine kinase inhibitor, EGFR-TKI），如第一代EGFR-TKI、吉非替尼、埃克替尼和厄洛替尼，第二代的阿法替尼以及第三代的奥西替尼在具有*EGFR*基因突变的非小细胞肺癌患者中的疗效已经得到肯定。国外研究发现吉非替尼可以抑制骨的重吸收，在发挥抗肿瘤治疗的同时也可以显著改善病理性骨折的发生，并且可以有更好的生存获益^[59-64]^。因此对携带*EGFR*基因敏感突变的非小细胞肺癌骨转移患者，EGFR-TKI可作为一线治疗方案，对于EGFR-TKI治疗后进展并且T790M突变阳性的患者推荐奥西替尼治疗。

（二）以棘皮动物微管相关蛋白样4-间变性淋巴瘤激酶（echinoderm microtubule-associated protein-like 4-anaplastic lymphoma kinase, *EML4-ALK*）融合基因和ROS1为靶点的酪氨酸酶抑制剂，中国非选择性非小细胞肺癌人群*EML4-ALK*融合基因发生率约为4%。克唑替尼是间变性淋巴瘤激酶（anaplastic lymphoma kinase, ALK）、MET和ROS-1的酪氨酸激酶的抑制剂，对有*EML4-ALK*融合基因的晚期非小细胞肺癌患者的疾病控制率可达60%-70%，已经成为继EGFR-TKI后又一种具有明确分子靶点和疗效预测标志的靶向药物。因此对于*ALK*或*ROS1*融合基因阳性的非小细胞肺癌患者，可选择克唑替尼治疗。克唑替尼耐药后可选择二代ALK抑制剂色瑞替尼。

（三）以VEGF为靶点的治疗：贝伐珠单抗是人源化抗血管内皮生长因子受体（vascular endothelial growth factor receptor, VEGFR）的单克隆抗体，可以与VEGFR结合，从而阻断VEGF与其受体结合，抑制肿瘤新生血管形成。贝伐珠单抗与化疗联合应用能够提高非鳞型非小细胞肺癌的治疗疗效并延长患者生存。贝伐珠单抗联合含铂双药化疗药物是目前晚期非鳞型非小细胞肺癌的标准一线治疗方案之一。在动物模型中可以观察到抗VEGFR靶向治疗可以有效治疗骨转移，有研究^[65]^发现治疗非鳞非小细胞肺癌贝伐珠单抗与化疗联合治疗骨转移有效率更高，可以延长骨转移中位进展时间，并且降低SREs的发生，因此也说明了贝伐珠单抗在增强化疗药的抗肿瘤活性同时还可以更好地发挥抑制骨转移的作用。

小分子抗VEGFR多靶点酪氨酸激酶抑制剂如安罗替尼也可用于肺癌骨转移患者的三线治疗。

#### 6.2.3 免疫治疗

免疫治疗药物为晚期肺癌的治疗提供了新的治疗选择。抗PD-1抗体Nivolumab和Pembrolizumab与T细胞的PD-1受体结合，抗PD-L1抗体可以与免疫细胞或肿瘤细胞的PD-L1受体结合，阻断PD-1/PD-L1通路对T细胞的抑制作用，从而激发抗肿瘤效应。临床研究中均显示无论抗PD-1抗体Nivolumab和Pembrolizumab或者抗PD-L1抗体Atezolizumab二线治疗晚期非小细胞肺癌均优于多西他赛化疗组，并且可以明显延长中位总生存时间^[66-70]^。因此目前也作为驱动基因阴性晚期肺癌的治疗推荐。

### 6.3 镇痛治疗^[71-76]^

对于肺癌骨转移疼痛的处理应采用综合治疗手段，即根据患者的病情、身体状况、疼痛部位及特点，应用恰当的止痛治疗手段，及早、持续、有效地消除疼痛，预防和控制药物的不良反应，提高患者生活质量。肺癌骨转移的镇痛治疗包括药物治疗和非药物治疗，后者包括放疗、手术和介入治疗。介入治疗是指神经阻滞术、神经松解术、经皮椎体成形术、神经损毁性手术及射频消融术等侵袭性干预措施。

#### 6.3.1 疼痛评估

对于肺癌骨转移疼痛患者，充分进行疼痛评估是合理、有效镇痛治疗的前提，应当遵循“常规、量化、全面、动态”的癌痛评估原则（参照《癌症疼痛诊疗规范（2018年版）》）。常规评估中需要注意爆发性发作的原因，如有无急需处理的病理性骨折及脊髓压迫等急症；量化评估中应重点评估最近24 h内患者最严重和最轻的疼痛程度以及平常情况的疼痛程度。通常应用数字分级法（numerical rating scale, NRS）、面部表情评估量表法及主诉疼痛程度分级法（verbal rating scales, VRS）三种方法（见附件1）；对疼痛病因和类型、疼痛发作情况（疼痛的部位、性质、程度、加重或减轻的因素）、止痛治疗情况、重要器官功能情况、心理精神情况、家庭及社会支持情况以及既往史（如精神病史、药物滥用史）等进行全面评估；对疼痛需进行全程、动态监测，评估癌痛患者的疼痛症状及变化情况。

#### 6.3.2 镇痛药物应用原则

对于肺癌骨转移患者疼痛药物治疗应结合世界卫生组织（World Health Organization, WHO）癌症三阶梯止痛治疗指导原则（见附件4）和《癌症疼痛诊疗规范（2018年版）》指导原则，肺癌骨转移疼痛患者药物止痛治疗遵循口服给药、按阶梯给药、按时给药、个体化给药和注意具体细节五大基本原则。

对于肺癌骨转移疼痛药物选择和治疗遵循《癌症疼痛诊疗规范（2018年版）》指导原则：即应当根据癌症患者疼痛的性质、程度、正在接受的治疗和伴随疾病等情况，合理地选择止痛药物和辅助镇痛药物，个体化调整用药剂量、给药频率，积极防治不良反应，以期获得最佳止痛效果，且减少不良反应。

#### 6.3.3 常用镇痛药物种类及注意事项

（1）非甾体类抗炎药物和对乙酰氨基酚：非甾体类抗炎药物具有止痛和抗炎作用。常见的有阿司匹林、布洛芬。有研究表明，选择性COX-2抑制剂可以减轻啮齿动物的骨痛行为，长期治疗也可减少肿瘤负荷和引起破骨细胞的破坏。常用选择性COX-2抑制剂有塞来昔布、依托考昔。对乙酰氨基酚具有镇痛和解热作用，但不具有抗炎作用。食品药品监督管理局（Food and Drug Administration, FDA）建议患者每日对乙酰氨基酚用量最多4 g，并在处方产品中每单位剂量（如每片、每粒胶囊）对乙酰氨基酚限制在325 mg，以减少因过量服用引起的严重肝损伤风险。常用于缓解轻度疼痛，或与阿片类药物联合用于缓解中、重度疼痛。

（2）阿片类药物：是中、重度癌痛治疗的首选药物。对于慢性癌痛治疗，推荐选择阿片受体激动剂类药物。长期使用阿片类止痛药时，首选口服给药途径，有明确指征时可选用透皮吸收途径给药，也可临时皮下注射用药，必要时可以自控镇痛给药。使用阿片类药物时需要注意剂量滴定方法、维持用药选择和不良反应管理，具体如下：

① 初始剂量滴定：对于初次使用阿片类药物止痛的患者，建议初始用药时选择短效阿片类止痛药，按照如下原则进行滴定：使用吗啡即释片进行治疗；根据疼痛程度，拟定初始固定剂量5 mg-15 mg，口服，*Q4h*或按需给药；用药后疼痛不缓解或缓解不满意，应于1 h后根据疼痛程度给予滴定剂量（见表 1），密切观察疼痛程度、疗效及药物不良反应。第1天治疗结束后，计算次日药物剂量：次日总固定量=前24 h总固定量+前日总滴定量。次日治疗时，将计算所得的次日总固定量分6次口服，次日滴定量为前24 h总固定量的10%-20%。依法逐日调整剂量，直到疼痛评分稳定在0分-3分。如果出现不可控制的药物不良反应，疼痛强度 < 4分，应考虑将滴定剂量下调10%-25%，并且重新评价病情。当用药剂量调整到理想止痛及安全的剂量水平时，可考虑换用等效剂量的长效阿片类止痛药；对于已经使用阿片类药物治疗疼痛的患者，可以根据患者的疗效和疼痛强度，参照表 1的要求进行滴定；对于疼痛病情相对稳定的患者，可以考虑使用阿片类药物缓释剂作为背景给药，在此基础上备用短效阿片类药物，用于治疗爆发性疼痛。阿片类药物缓释剂的剂量调整参考表 1。但需要注意区别爆发性疼痛是否因病理性骨折、脊髓神经压迫所致，此类患者需要骨科等科室进行处理。

**1:** 剂量滴定增加幅度参考标准

疼痛强度（NRS）	剂量滴定增加幅度
7-10	50%-100%
4-6	25%-50%
2-3	≤25%

② 维持用药。在我国常用的长效阿片类药物有吗啡缓释片、羟考酮缓释片和芬太尼透皮贴剂等。在应用长效阿片类药物期间，应备用短效阿片类止痛药，用于爆发性疼痛。当患者因病情变化，长效止痛药物剂量不足时，或发生爆发性疼痛时，立即给予短效阿片类药物，用于解救治疗及剂量滴定。解救剂量为前24 h用药总量的10%-20%。每日短效阿片解救用药次数≥3次时，应当考虑将前24 h解救用药换算成长效阿片类药按时给药。

阿片类药物之间的剂量换算，可参照换算系数表（见表 2）。换用另一种阿片类药时，仍然需要仔细观察病情变化，并且个体化滴定用药剂量。

**2:** 阿片类药物剂量换算表

药物	非胃肠给药	口服	等效剂量
吗啡	10 mg	30 mg	非胃肠道：口服=1:3
可待因	130 mg	200 mg	非胃肠道：口服=1:1.2吗啡（口服）：可待因（口服）=1:6.5
羟考酮	10 mg		吗啡（口服）：羟考酮（口服）=l.5-2:1
芬太尼透皮贴剂	25 *μ*g/h（透皮吸收）		芬太尼透皮贴剂*μ*g/h，*q72h*剂量=1/2×口服吗啡mg/d剂量

如需减少或停用阿片类药物，应该采用逐渐减量法，一般情况下阿片剂量可按照每天剂量减少10%-25%，直到每天剂量相当于30 mg口服吗啡的药量，再继续服用2 d后即可停药。

③ 不良反应防治。阿片类药物的常见不良反应包括便秘、恶心、呕吐、嗜睡、瘙痒、头晕、尿潴留、谵妄、认知障碍以及呼吸抑制等。除了便秘之外，这些不良反应大多是暂时性的或可以耐受的。应把预防和处理阿片类止痛药不良反应作为止痛治疗计划和患者宣教的重要组成部分。对于便秘的患者，除关注不良反应之外，需要鉴别有无脊髓压迫等。

（3）双膦酸盐：可改善肿瘤骨组织的酸性微环境，导致骨溶解减少，减轻癌痛（详见6.1骨改良药物）。

（4）辅助镇痛用药：主要包括抗惊厥类药物、抗抑郁类药物、皮质激素、N-甲基-D-天冬氨酸受体（N-methyl-D-aspartate receptor, NMDAR）拮抗剂和局部麻醉药等。辅助用药的种类选择和剂量调整，也需要个体化对待。常用于神经病理性疼痛的辅助药物：

① 抗惊厥类药物：用于神经损伤所致的撕裂痛、放电样疼痛及烧灼痛。

② 三环类抗抑郁药：用于中枢性或外周神经损伤所致的麻木样痛、灼痛，该类药物也可以改善心情、改善睡眠。

③ 激素类药物：对于肺癌脊柱转移有脊髓压迫症状的患者具有较好的治疗效果，常用药物包括甲基强的松龙、地塞米松等。

肺癌骨转移疼痛镇痛药物选择和使用具体见附件5，但在肺癌骨转移患者出现爆发痛、尿潴留、感觉异常的患者中，需要严格鉴别是否为病理性骨折及脊髓压迫所致。

癌痛综合治疗

1.癌痛综合评估（见附件1）

2.姑息性抗癌治疗及全身性非阿片类/阿片类镇痛药物

3.全身阿片类药物治疗弊大于利时，考虑非侵袭性干预措施^[3-5]^：恰当姑息性抗癌治疗；加用非阿片类药物；加用辅助药物；应用认知和行为干预措施；借助矫形疗法、其他物理疗法和社会心理干预

4.全身阿片类药物治疗弊大于利时，考虑侵袭性干预措施：区域性止痛技术；神经阻滞术；神经切断术

5.若上述方法无效时，应用镇静剂等辅助药物协助处理顽固性疼痛

### 6.4 放射治疗

放射治疗是肺癌骨转移有效的治疗方法之一，能够减轻或消除症状、改善生活质量、延长生存，还能预防病理性骨折和脊髓压迫的发生及缓解脊髓压迫症状。放射治疗包括外照射和放射性核素治疗两类。

#### 6.4.1 体外放射治疗

体外放射治疗是肺癌骨转移姑息性放疗的首选方法，对经化疗和双膦酸盐治疗后仍无法缓解的顽固性疼痛、椎体不稳、即将发生病理性骨折和脊髓压迫症的患者，局部放疗可迅速有效地缓解骨破坏和软组织病变导致的疼痛。对于长骨骨折患者，放疗可有效控制疼痛，并有可能促进骨折愈合。随着放疗技术的发展，立体定向放射治疗（stereotactic body radiotherapy, SBRT）的出现给肺癌骨转移患者带来了新的希望。SBRT可显著提高患者的局部控制率^[77]^，并可显著缓解骨转移引起的疼痛症状^[78, 79]^，给患者带来更好的生活质量。

由于双膦酸盐阻止肿瘤细胞由G_2_期和M期向S期转换，延长肿瘤细胞在放疗敏感的细胞周期的时段，故可常规联用双膦酸盐以增强骨转移灶对放疗的敏感性^[80, 81]^。

（1）治疗前评估患者全身症状及其他部位肿瘤情况：

① 患者全身状况及其他部位肿瘤情况评价：

1）PS评分 < 2分，全身病变稳定：较高剂量较长时间或对寡转移病灶采用SBRT技术，尽量提高局部控制率；

2）PS评分2分-3分，其他部位进展：较低剂量较短时间，姑息缓解疼痛为主。

骨转移部位：

1.承重骨即使无症状亦可预防照射，减少骨不良事件发生；

2.非承重骨其他治疗手段缓解疼痛症状无效、影响功能，尽早局部放疗。

（2）体外放射治疗适应证：①有疼痛症状的骨转移灶，缓解疼痛及恢复功能；②选择性地用于负重部位骨转移的姑息性放疗（如脊柱或股骨转移）^[82, 83]^；③骨寡转移SBRT治疗^[84-86]^。

骨痛需要放疗的SREs定义：①非承重骨的骨转移，伴骨痛（VAS 4分），经中度止痛药无效而接受放疗属于SREs；②承重骨骨转移，伴疼痛（VAS 4分）接受放疗属于SREs；③承重骨骨转移无疼痛，但有明显骨质破坏而接受放疗则属于伴随治疗。

（三）体外放射治疗常用剂量及分割方法：①300 cGy/次，共10次；②400 cGy/次，共6次；③400 cGy/次，共5次；④800 cGy/次，单次照射（顽固性疼痛、已发生或即将发生的病理性骨折的患者，推荐剂量为8 Gy/次-10 Gy/次）；⑤对于寡转移或者形成软组织肿块的骨转移病灶可适当提高放疗剂量，包括采用SBRT技术。

（四）单次放射及多次放射的适应证：

单次放射适用于：①非中线骨转移；②行动不便急需解决骨痛的患者。

多次放射适用于：①因其他治疗止痛无效因疼痛需要放疗的骨转移患者；②有骨折风险患者。对大多数无椎体骨或重要结构骨转移的初治骨转移瘤患者，可推荐单次大剂量放疗^[83]^。但再放疗和病理性骨折的发生率高于分次放疗。推荐单次放疗用于活动及搬运困难的晚期肺癌骨转移患者^[87]^。

（五）疗效评价：①疼痛改善程度：完全缓解：指疼痛明显减轻或基本消失，恢复正常活动，基本可以不用止痛药。部分缓解：指疼痛减轻，止痛药使用明显减少，因骨转移所导致的功能障碍部分缓解；无效：疼痛略减轻或无明显缓解，止痛药物剂量不能减少。②影像学检查。

#### 6.4.2 放射性核素治疗

放射性核素治疗放射性核素治疗是肺癌骨转移的一种有效的治疗手段。放射性核素治疗应严格掌握适应证，不能优先选择。主要是由于部分患者放射性核素治疗后会出现明显的骨髓抑制且恢复较慢，影响化疗等后续全身治疗。因此，放射性核素治疗前应影像学确认，多学科共同评估，为患者选择合适的治疗方案及恰当的治疗时机。

（一）目前骨转移癌放射性核素治疗的常用药物是^89^Sr。^89^Sr是骨转移内科放射治疗中最常用的核素药物，半衰期50.5 d，骨组织中射程约3 mm，发射纯β射线，其体内代谢特点与钙相似。肿瘤细胞破坏骨组织，导致成骨修复活跃，骨组织代谢增高，浓聚大量的^89^Sr。病变骨组织与正常骨组织的摄取比为2:1-25:1；^89^Sr在正常骨的有效半衰期为14 d，在肿瘤骨转移灶内的有效半衰期长于50 d，因此可使病灶获得较高的辐射吸收剂量，所以能获得较好疗效。

（二）适应证及禁忌证、适应证：（1）诊断明确的多发性骨转移肿瘤，^99^Tcm-MDP骨显像证实骨转移病灶处有浓聚。即使X线检查为溶骨性病灶，只要骨显像该病灶浓聚^99^Tcm-MDP，^89^Sr治疗就可能获得疗效。（2）原发性骨肿瘤未能手术切除或术后残留病灶或伴骨内多发转移者，^99^Tcm-MDP骨显像证实病灶处有浓聚。（3）治疗前1周内的血红蛋白 > 90 g/L，白细胞≥3.5×10^9^/L，血小板≥80×10^9^/L。禁忌证：（1）绝对禁忌证。妊娠或哺乳期患者。（2）相对禁忌证。由于放射性药物可能产生的骨髓毒性，血细胞计数低至一定范围是使用^89^Sr的相对禁忌证，但目前还未明确定义相关指标准确的低限。在没有合并慢性弥漫性血管内凝血（disseminated intravascular coagulation, DIC）的情况下，权衡利弊，血细胞计数的下限可放宽至：白细胞总数 > 2.4×10^9^/L，血小板≥60×10^9^/L。血肌酐 > 180 μmol/L和（或）肾小球滤过率（glomerular filtration rate, GFR） < 30 mL/min的患者应避免接受^89^Sr治疗。脊髓压迫和病理性骨折急性期患者应避免单独接受^89^Sr治疗，也不宜用于预期生存短于8周的患者。

（三）注意事项：^89^Sr治疗前后行局部放疗是安全的，但治疗前后3个月内应避免行大野放疗（半身放疗）；在^89^Sr治疗前4周-8周内、治疗后6周-12周内应停用具有长效骨髓抑制作用的化疗药物；DIC是^89^Sr治疗后引起严重血小板减少症的危险因素，在^89^Sr治疗前应行凝血功能检测以排除亚临床DIC，尤其应注意近期有血小板急剧降低的患者；应排除非骨肿瘤导致的骨痛患者，如脊髓压迫、肿瘤组织压迫等；如受肿瘤侵犯的骨骼有50%以上的骨质破坏（尤其是四肢骨），或者伴有病理性骨折，应避免单独使用^89^Sr治疗。

（四）常用剂量及方法：常用剂量为1.48 MBq/kg-2.22 MBq/kg，成人一般为148 MBq/次。临床结果表明，低于1.11 MBq/kg缓解疼痛的疗效不好，过大剂量不但加重经济负担和不良反应，而且疗效并不随剂量的增加而提高。给药方法：一次静脉缓慢注射给药（1 min-2 min）。应先建立静脉通道便于注射后用生理盐水冲洗，避免^89^Sr注射液渗漏。发生渗漏后局部热敷可加快药物吸收，降低局部辐射剂量。第一次治疗疗效好，骨痛未完全消失或复发，可重复治疗；^89^Sr重复治疗间隔3个月或更长时间；对于第一次注射后无反应的患者，第二次治疗50%的患者可获得疗效。

（五）疗效评价：^89^Sr治疗有效的患者治疗后2 d-7 d约64%的患者出现疗效，治疗后4周90%的患者出现疗效，疼痛缓减一般可持续3个月，报道疼痛缓减持续最长时间可达15个月。经^89^Sr治疗后患者生活质量可获得显著改善，行为能力评分可提高20%以上。5%-10%的患者在^89^Sr注射后出现短暂的疼痛加重，称之为反跳痛或称骨痛闪烁现象，一般发生在注射后3 d-6 d，持续约2 d-7 d，通常预示可获得较好的疗效，如果出现疼痛加重，可加大止痛药用量或将止痛药升级。^89^Sr治疗后患者的部分病灶^99^Tcm-MDP骨显像的放射性摄取降低；X线检查显示病灶缩小，溶骨性病灶有再钙化征象；部分患者骨转移灶数目减少，有的患者甚至骨转移灶完全消失^[88]^。

### 6.5 外科治疗

由于肺癌整体治疗的进步，其中位生存期较前不断提高，因此骨转移瘤相关SREs发生率亦增高，很多情况下，如不对骨转移灶进行外科干预，患者生存质量将受到明显影响^[89]^。肺癌骨转移往往导致骨强度下降、进而患者的运动系统功能受损^[90]^。“生命在于运动”强调了运动系统功能对于患者生命的重要意义，外科治疗的意义在于恢复运动系统功能，因此，肺癌骨转移的外科治疗也与原发病变的治疗显得同样重要。

#### 6.5.1 外科治疗的主要目的

包括：1）获得骨转移病灶的组织学诊断，便于肿瘤的进一步内科治疗；2）缓解疼痛；3）预防或治疗骨折^[91]^；4）提高生存质量；5）减少或避免运动系统功能受损所引发的并发症，间接提高患者生存期。

#### 6.5.2 外科治疗的适应证

包括^[92]^：1）预计患者可存活3个月以上；2）全身状况好，能够耐受手术创伤及麻醉；3）预计外科治疗后，患者可获得较术前更好的生活质量，甚至能够立即恢复运动系统功能，有助于进一步治疗和护理；4）预计原发肿瘤治疗后有较长的无瘤期；5）全身治疗有效，但局部出现症状者；6）孤立的骨转移病灶；7）病理骨折风险高者；8）已发生脊柱不稳定或脊髓受压或者高风险者。

#### 6.5.3 外科治疗时机

1）有恶性肿瘤病史，影像学及组织学检查为单发骨转移者；2）负重骨出现平片可见的骨破坏；3）保守治疗后，骨破坏仍继续加重的患者；4）保守治疗后，疼痛仍继续加重的患者；5）保守治疗后，运动系统功能仍不能恢复者；6）已经出现病理骨折的患者；7）有神经压迫症状者；8）脊柱溶骨性破坏，出现截瘫危险性大的患者；9）放、化疗治疗不敏感骨转移灶，如肾癌骨转移等。

#### 6.5.4 不同部位外科治疗的手术适应证

（一）负重长管状骨：1）即将发生骨折；2）已发生骨折；3）病变直径 > 2.5 cm；4）病变 > 50%皮质；5）完全溶骨；6）负重下疼痛；7）放疗后疼痛。

需要注意：应在病理骨折发生前进行外科干预，使患者免受不必要的骨折痛苦。预防性内固定的治疗比已发生骨折的治疗要简单和安全。应用Mirels评分系统（详见表 3）可有效评估病理骨折风险^[93]^，从而指导预防性内固定的实施，评分7分及以下可暂时不考虑手术，而评分7分以上者，应考虑手术治疗。

**3:** Mirels长骨病理骨折风险评估模型

分值	解剖部位	性质	大小	疼痛
1	上肢	成骨性	< 1/3	轻度
2	下肢（非小粗隆部位）	混合性	1/3-2/3	中度
3	小粗隆部位	溶骨性	> 2/3	功能性

（二）脊柱：1）神经功能受损；2）脊柱不稳定；3）即将发生骨折；4）疼痛^[94]^。

（三）骨盆：1）髋臼即将或已发生病理骨折；2）顽固性疼痛；3）对侧即将发生骨折而需外科治疗^[95]^。

#### 6.5.5 外科禁忌证

对于下列因素应考虑非手术治疗：1）预计生存期短于3个月；2）全身广泛骨破坏；3）涉及多器官广泛转移；4）全身状况差，有手术禁忌证。

#### 6.5.6 外科治疗的基本思想及关键点

（1）肺癌骨转移瘤的治疗需多学科协作，骨科医师、肿瘤内科医师及放疗科医师应分工明确。在制定治疗方案时应考虑的因素包括：预期寿命、肿瘤的类型和分期、有无内脏转移、Karnofsky和Burchenal患者状况评分、发现原发灶至出现转移灶的时间、病理骨折的风险以及对化疗、激素疗法和放疗敏感程度的预测。外科治疗的基本思想：无需期待骨愈合，固定要即刻坚定。

（2）长管状骨转移癌外科治疗关键点^[96]^：1）内置物坚强、稳定；2）治疗包括所有骨强度降低区；3）尽可能切除肿瘤；4）内置物寿命长于患者寿命。

（3）脊柱转移瘤外科治疗关键点：1）病变多位于椎体，可采用前入路；2）尽量去除肿瘤，彻底解除对脊髓的压迫；3）避免单纯后路椎板减压术，这可能会加重脊柱不稳定；4）前路重建纠正后突畸形，后路重建维护脊柱稳定性；5）椎体成形术并不完全适于椎体转移瘤的治疗，风险大且效果不确定^[97]^。

（4）骨盆转移瘤外科治疗关键点：1）未累及髋臼的髂骨病变，应用内固定及骨水泥加强应力传导区；2）累及髋臼的髂骨病变，可考虑行全髋关节置换，并应用内固定及骨水泥加强应力传导区；3）非应力传导区病变（耻、坐骨），可行单纯切除。

随着肺癌整体治疗的明显提高和骨转移瘤多学科综合治疗模式的发展，我们需要提高多学科对骨转移瘤外科治疗的认识，同时也要提高外科医生的肿瘤学知识。建立较为完善的骨转移癌外科治疗综合评估系统，在多学科综合治疗指导下为患者选择恰当的综合治疗方式，显得尤为重要^[98]^。肿瘤内科医生提供的全身治疗，骨科医师提供的外科治疗，以及放疗科医师的放疗支持，需要进行综合考虑，合理综合应用将有助于提高患者的生存质量及总生存期^[99]^。

### 6.6 介入治疗

随着微创理念的更新及医疗技术的进步，多种影像引导下的微创介入治疗技术在肺癌骨转移局部控制、减轻疼痛、改善生活质量方面的应用也逐渐成熟。目前常用手段主要包括消融治疗（射频消融、微波消融、激光消融、冷冻消融以及高强度聚焦超声等）、骨成形术、近距离治疗（放射性粒子植入）等。因其具有操作简便、创伤微小、安全性高、副作用少、恢复快速等优点而为无法耐受或不愿接受其他治疗手段的患者提供了另一种选择。

#### 6.6.1 消融治疗

肺癌骨转移的消融治疗是利用热产生的生物学效应直接导致病灶组织中的肿瘤细胞发生不可逆损伤或凝固性坏死的一种精准微创的治疗技术^[100]^。

其适应证及禁忌证如下^[101, 102]^：

适应证：（1）因全身情况差不能耐受手术或拒绝手术、放疗后复发者；（2）病灶数目≤5个，病灶边缘距脊髓、神经等重要结构≥1 cm；（3）中重度疼痛，疼痛评分≥4分。

禁忌证：（1）严重的肝肾心肺脑功能不全者；（2）严重的出血倾向，血小板计数 < 50×10^9^/L。

文献报道^[103, 104]^消融术后1周，患者疼痛感受减轻，生活质量改善。随访1个月时92%的患者疼痛缓解，6个月81%的患者疼痛缓解。有学者^[105]^使用冷冻消融、聚焦超声治疗骨转移引发疼痛方面也收到了与射频消融类似的效果。

#### 6.6.2 骨成形术

骨成形术（osteoplasty）是经穿刺通道将甲基丙烯酸甲酯（PMMA，又称骨水泥）注入病灶内，从而达到稳定骨结构、缓解疼痛和局部控制肿瘤的介入治疗技术。包括经皮椎体成形术、后凸成形术、全身不规则骨及四肢长骨骨水泥灌注术等。

其适应证及禁忌证如下^[106]^：

适应证：经皮骨成形术适用于各种溶骨性骨原发肿瘤或骨转移瘤。

禁忌证：（1）严重神经系统疾患或全身情况差难以耐受手术及麻醉；（2）难以纠正的凝血障碍；（3）肿瘤侵及重要的脏器、神经、血管；（4）活动性感染；（5）病变有5处以上转移灶或广泛性弥漫性转移。

文献^[107, 108]^报道骨成形术可显著缓解患者骨转移部位的疼痛且复发率低。有学者^[109]^通过对肺癌椎体转移病例行椎体成形术后3个月、6个月、12个月及≥18个月的随访，发现局部控制率分别为100%、97.2%、93.05%及85.71%，显示局控效果显著。

#### 6.6.3 近距离治疗

近距离治疗是指将密封的固体辐射源置入人体病灶内部进而治疗肿瘤的一种微创治疗方法，我国用于肺癌骨转移的放射源一般为125I。放射源产生的射线进入组织后可以通过产生氧自由基来杀死肿瘤细胞，同时可以通过破坏肿瘤细胞细胞核内的DNA和抑制有丝分裂来杀死肿瘤细胞。射线持续的照射可以产生累积损伤，从而减轻肿瘤组织引起的生物学疼痛^[110]^。

其适应证及禁忌证如下^[111]^：

适应证：（1）拒绝或不适合手术切除、拒绝外放射治疗，手术或外放射治疗后复发者，肿瘤大小≤7 cm；（2）有合适的穿刺路径；（3）身体条件良好（Karnofsky评分 > 70分）；（4）能耐受放射性粒子植入手术；（5）预计寿命≥3个月。

禁忌证：（1）严重出血倾向，血小板计数 < 50×10^9^/L，严重的凝血功能障碍（凝血酶原时间 > 18 s），凝血酶原活动度≤40%，抗凝治疗和/或抗血小板药物停用小于1周；（2）肿瘤溃疡；（3）无合适的穿刺路径；（4）预计划靶区剂量达不到处方剂量设计要求。

文献^[112, 113]^报道近距离治疗的局部控制率很高且术后2个月-12个月患者骨转移的疼痛明显减轻，缓解率接近100%。生活质量（包括睡眠质量、饮食、精神状态、KPS评分）较治疗前亦有明显改善。同时因近距离治疗具有局部肿瘤完全辐射而周围正常组织剂量低、瘤体内剂量分布均匀、可重复植入等优点，是一种局部控制肿瘤、减轻疼痛的有效治疗方法。

### 6.7 心理支持治疗

根据骨转移姑息治疗的基本原则，应针对骨转移及其相关并发症提供最佳支持治疗和症状治疗，需要肿瘤临床医生与心理精神科医生建立好一个多学科合作团队。心理精神科医生应对患者进行心理精神症状的评估，达到临床诊断意义的心理痛苦需要精神心理医生进行相应诊疗，改善患者心理精神痛苦。

#### 6.7.1 与肺癌及骨转移相关的心理精神症状的处理

有研究^[114]^提示在癌症患者中，心理痛苦的总患病率为35.1%，不同类型癌症间存在差异，其中肺癌患者的心理痛苦患病率最高，达到43.4%。国外有一项研究^[115]^调查了门诊就诊的肺癌患者，62%存在明显的心理痛苦，其预测因素有年轻、疼痛、疲劳、焦虑和抑郁。肺癌患者面临最多的是抑郁情绪，国内外多项调查^[116, 117]^得出结论，肺癌患者的抑郁患病率是所有恶性肿瘤中最高的，包括重度抑郁的患病率。根据患者心理精神症状的临床诊断及严重程度进行相应的干预治疗。没有达到临床诊断的心理痛苦可由临床医护人员给予相应的心理支持和患者教育，以降低患者对疾病进展的恐惧和担心程度，适应疾病的状态。达到临床诊断意义的心理痛苦需要精神心理医生进行会诊和合作指导。

#### 6.7.2 心理治疗的主要内容及方法

心理社会干预可以有效缓解癌症患者的心理痛苦并改善总体生活质量。支持性治疗、认知-行为治疗、家庭治疗以及CALM（Managing Cancer And Living Meaningfully）治疗是比较常用的心理治疗方式。癌症患者焦虑、抑郁的主要临床表现及治疗方法见附件6。

#### 6.7.3 焦虑抑郁障碍的治疗

包含精神药物治疗和心理治疗。对于轻到中度焦虑抑郁障碍患者可选择心理治疗、支持治疗、行为治疗等，而重度焦虑抑郁障碍则首选药物治疗，大多数情况下，可选择联合治疗^[118]^。常用癌症患者的主要抗焦虑和抗抑郁药物用法及注意事项参见附件7和附件8。

### 6.8 肺癌骨转移治疗流程

见图 2。

**2:**
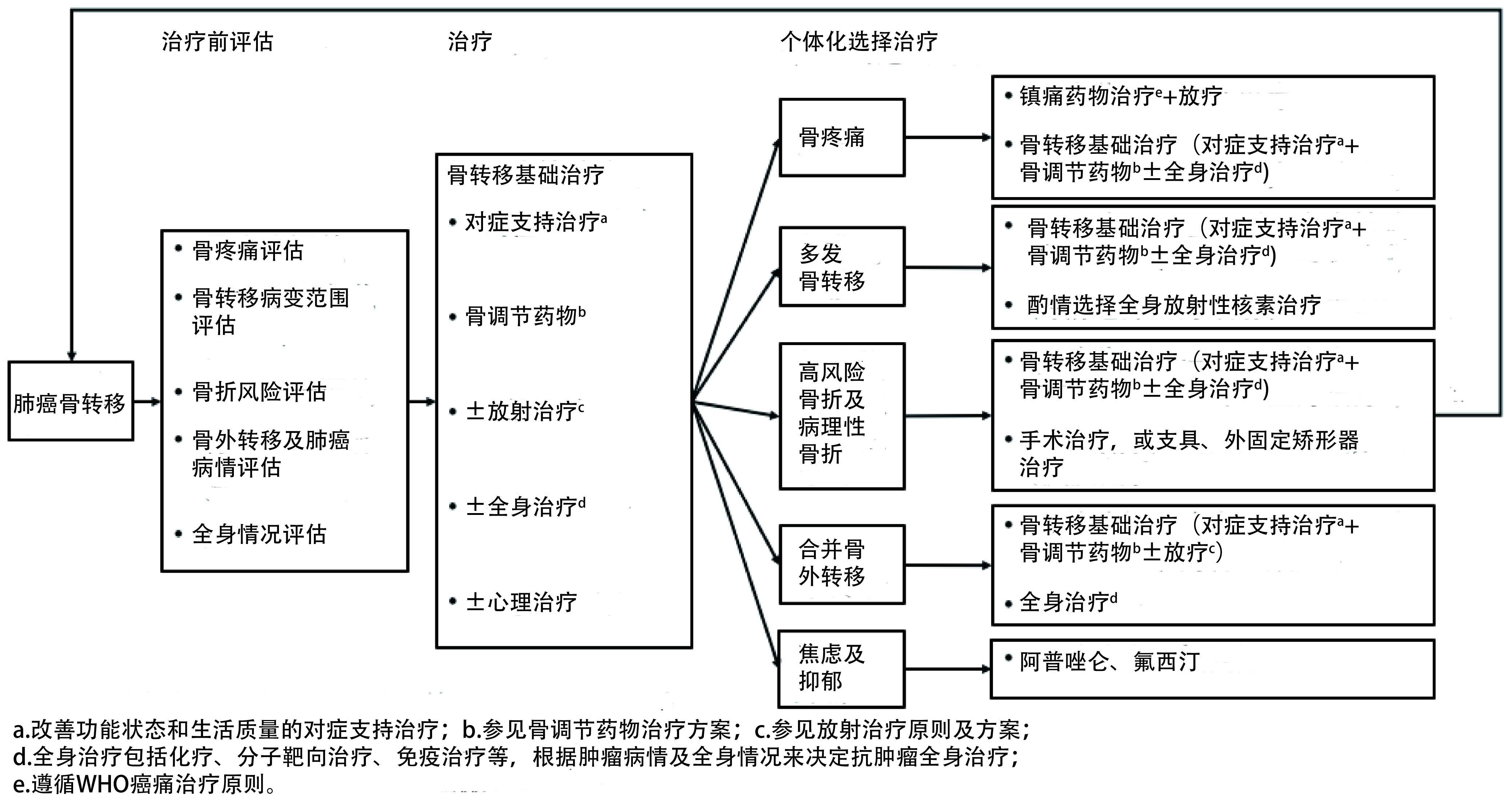
肺癌骨转移治疗流程

## 7 诊疗推荐

### 7.1 诊断推荐

对怀疑有骨转移的肺癌患者推荐进行以下检查：

1 放射性核素骨扫描（ECT）检查或PET-CT检查

2 ECT检查阳性的部位行X线平片

3 ECT检查阳性的部位行CT及/或MRI检查

4 血常规、肌酐、肝功能、电解质、血清钙等生化指标检查

### 7.2 化疗、分子靶向治疗及免疫治疗推荐

含铂化疗方案或联合贝伐珠单抗是非小细胞肺癌骨转移的标准一线治疗方案，对携带*EGFR*敏感突变或者*ALK*、*ROS-1*融合基因阳性患者，推荐尽早使用EGFR-TKI或克唑替尼治疗，对于驱动基因阴性晚期肺癌，免疫治疗也是推荐的选择。

### 7.3 放射治疗推荐

骨转移姑息性放射治疗及选择：

1.体外照射：局部或区域放疗，骨转移放射治疗的常规放疗方法体外放射治疗适应证：①有疼痛症状的骨转移灶，缓解疼痛及恢复功能；②选择性地用于负重部位骨转移的姑息性放疗（如脊柱或股骨转移）；③骨寡转移SBRT治疗。

体外放疗常用剂量及分割方法：（选择下列方法之一）：①300 cGy/次，共10次；②400 cGy/次，共6次；③400 cGy/次，共5次；④800 cGy/次，单次照射（顽固性疼痛、已发生或即将发生的病理性骨折的患者，推荐剂量为8 Gy/次-10 Gy/次）；⑤对于寡转移或者形成软组织肿块的骨转移病灶可适当提高放疗剂量，包括采用SBRT技术。

放疗可常规联合双膦酸盐治疗。

2.放射性核素：全身性内照射放疗，是肺癌骨转移的一种有效的治疗手段。

目前骨转移癌放射性核素治疗常用药物包括：^89^Sr和^153^Sm，^153^Sm已较少应用。

适应证：①经临床、CT或MRI、全身骨显像和病理确诊多发骨转移肿瘤，尤其是前列腺癌、乳癌和肺癌骨转移患者且全身骨ECT显像病灶处有放射性浓聚；②骨转移肿瘤患者伴骨痛；③白细胞≥3.5×10^9^/L，血小板≥80×10^9^/L。

禁忌证：①骨显像示转移灶仅为溶骨型冷区；②严重骨髓、肝肾功能障碍患者；③近期（6周内）进行过细胞毒素治疗患者。

常用剂量及方法：^89^Sr常用剂量为1.48 MBq/k-2.22 MBq/kg，成人一般为148 MBq/次，3个月-6个月后可重复应用。给药方法：一次静脉缓慢注射给药（1 min-2 min）。

注意：该治疗发生骨髓抑制风险较高，且恢复较慢（约12周）。

### 7.4 外科治疗推荐

1. 负重长管状骨手术适应证：1）即将发生骨折；2）已发生骨折；3）病变直径>2.5 cm；4）病变>50%皮质；5）完全溶骨；6）负重下疼痛；7）放疗后疼痛。

2. 脊柱：1）神经功能受损；2）脊柱不稳定；3）即将发生骨折；4）疼痛。

3. 骨盆：1）髋臼即将或已发生病理骨折；2）顽固性疼痛；3）对侧即将发生骨折而需外科治疗。

4. 适应证：1）预计患者可存活3个月以上；2）全身状况好，能够耐受手术创伤及麻醉；3）预计外科治疗后，患者可获得较术前更好的生活质量，甚至能够立即恢复运动系统功能，有助于进一步治疗和护理；4）预计原发肿瘤治疗后有较长的无瘤期；5）全身治疗有效，但局部出现症状者；6）孤立的骨转移病灶；7）病理骨折风险高者；8）已发生脊柱不稳定或脊髓受压、或者高风险者。

5. 禁忌证：对于下列因素应考虑非手术治疗: 1）预计生存期短于3个月；2）全身广泛骨破坏；3）涉及多器官广泛转移；4）全身状况差，有手术禁忌证。

### 7.5 镇痛治疗用药推荐

**Table:** 常用三阶梯镇痛药物

阶梯	药物
第一阶梯	对乙酰氨基酚、阿司匹林、布洛芬、吲哚美辛、萘普生、双氯芬酸钠、塞来昔布
第二阶梯	可待因、布桂嗪、曲马多、泰勒宁
第三阶梯	吗啡、硫酸吗啡缓释片、盐酸羟考酮缓释片、芬太尼透皮贴剂、美沙酮

### 7.6 双膦酸盐治疗推荐

双膦酸盐药物治疗骨转移的用法用量

1. 氯膦酸盐片剂1, 600 mg/d，口服给药；或氯膦酸盐针剂300 mg/d，静脉注射，>2 h持续5 d，之后换成口服给药；

2. 帕米膦酸盐90 mg，静脉注射>2 h，每3周-4周重复一次；

3. 唑来膦酸盐4 mg，静脉注射>15 min，每3周-4周重复一次；

4. 伊班膦酸盐6 mg，静脉注射>15 min，每3周-4周重复一次；伊班膦酸负荷疗法6 mg，静脉注射>15 min，连续3 d，持续每3周-4周重复一次。

5. 因卡膦酸二钠10 mg，65周岁以上患者推荐剂量为一次5 mg，静脉注射2 h-4 h，每3周-4周重复一次。

双膦酸盐适应证

1. 适应证：①骨转移引起的高钙血症；②骨转移引起的骨痛；③ECT异常，X线或CT、MRI证实骨转移；④ECT异常，X线正常，但CT或MRI显示骨破坏；⑤无骨痛症状，但影像学诊断为骨破坏。

2. 下列情况不推荐使用双膦酸盐：①ECT异常，X线正常，CT或MRI也未显示骨破坏；②存在骨转移风险（LDH或ALP增高）的患者。

双膦酸盐停药指征

1. 用药过程中检测到与治疗相关的严重不良反应；

2. 继续用药不能获益。

### 7.7 心理干预推荐^[119]^

**Table:** 

轻度焦	临床医护人员给予一些支持性干预
虑抑郁	·说明诊断、治疗选择和副作用 ·确保患者理解疾病和治疗选择 ·推荐适合的患者读物·告诉患者过渡期会更容易感受到痛 ·承认有痛苦 ·建立信任 进行心理社会评估，明确与癌症照料相关的心理、社会、支持性交流、心理教育、应激管理等。
中度焦	除了上述干预之外，提供心理和/或精神药物治疗；
虑抑郁	心理干预依据患者的特点、病程阶段以及癌症相关问题开展；心理教育或认知-行为治疗的问题解决方法适用于疾病的早期阶段，支持-表达性治疗有助于晚期癌症患者
重度焦	·药物治疗*联合心理社会干预；需提供专业的心理社会服务
虑抑郁	·镇痛药 ·抗焦虑药 ·安眠药 ·抗抑郁药 ·支持小组和/或个体咨询 ·家庭支持和咨询 ·放松、冥想、创造性治疗
*抗抑郁药应根据患者的症状及药理因素(包括不良反应、耐受性以及潜在的药物交互作用)进行选择。在抗抑郁治疗的第一周内应评估药物不良反应，若出现不良反应，应停药或换药^[118, 119]^。	

**附件**：

1. 疼痛量化评估常用方法; 2. 患者一般情况评估 (Karnofsky评分); 3. ZPS评分; 4. WHO癌痛三阶梯止痛; 5. 常见癌痛治疗药物表; 6. 焦虑、抑郁主要临床表现及治疗; 7. 癌症患者抗焦虑药物; 8. 癌症患者抗抑郁药物

**专家组成员**：

**发起人**：支修益（首都医科大学肺癌诊疗中心，首都医科大学宣武医院胸外科），王洁（中国医学科学院肿瘤医院肿瘤内科）

**执笔**： 董智（北京肿瘤医院暨北京市肿瘤防治研究所，恶 性肿瘤发病机制及转化研究教育部重点实验室 胸部肿 瘤内一科）；赵军（北京肿瘤医院暨北京市肿瘤防治研究 所，恶性肿瘤发病机制及转化研究教育部重点实验室 胸 部肿瘤内一科）；柳晨（北京肿瘤医院暨北京市肿瘤防治 研究所，恶性肿瘤发病机制及转化研究教育部重点实验 室 介入治疗科）；唐丽丽（北京肿瘤医院暨北京市肿瘤防 治研究所，恶性肿瘤发病机制及转化研究教育部重点实 验室 康复科）；汪艳（北京肿瘤医院暨北京市肿瘤防治 研究所，恶性肿瘤发病机制及转化研究教育部重点实验 室 康复科）；李梓萌（北京肿瘤医院暨北京市肿瘤防治 研究所，恶性肿瘤发病机制及转化研究教育部重点实验 室 康复科）；陈麦林（北京肿瘤医院暨北京市肿瘤防治 研究所，恶性肿瘤发病机制及转化研究教育部重点实验 室 医学影像科）；李囡（北京肿瘤医院暨北京市肿瘤防治 研究所，恶性肿瘤发病机制及转化研究教育部重点实验 室 核医学科）；郭锐（北京肿瘤医院暨北京市肿瘤防治研 究所，恶性肿瘤发病机制及转化研究教育部重点实验室 核医学科）；杨丹（北京肿瘤医院暨北京市肿瘤防治研究 所，恶性肿瘤发病机制及转化研究教育部重点实验室 放 射治疗科）；石安辉（北京肿瘤医院暨北京市肿瘤防治研 究所，恶性肿瘤发病机制及转化研究教育部重点实验室 放射治疗科）；李东（北京新里程肿瘤医院介入治疗科）； 潘峰（北京大学人民医院影像科）；孙昆昆（北京大学人民 医院病理科）；李琳（北京医院肿瘤内科，国家老年医学 中心）；李旭（北京医院肿瘤内科，国家老年医学中心）； 朱翔（北京大学第三附属医院病理科）；姜亮（北京大学 第三附属医院骨科）；曹宝山（北京大学第三附属医院肿 瘤化疗与放射病科）；易福梅（北京大学第三附属医院肿 瘤化疗与放射病科）；胡牧（首都医科大学附属友谊医院 胸外科）；牛晓辉（北京积水潭医院骨肿瘤科）；徐海荣 （北京积水潭医院骨肿瘤科）；段建春（中国医学科学院 肿瘤医院肿瘤内科）

**参与专家**：周彩存（上海市肺科医院肿瘤科），陆舜（上海市肺部肿瘤临床医学中心，上海交通大学附属胸科医院），刘晓晴（解放军总医院第五医学中心肿瘤科），张兰军（中山大学附属肿瘤医院胸外科），王长利（天津市肿瘤医院肺部肿瘤科），陈军（天津医科大学总医院肺部肿瘤外科），杨跃（北京大学肿瘤医院暨北京市肿瘤防治研究所胸外二科，恶性肿瘤发病机制及转化研究教育部重点实验室）

附件1  疼痛量化评估常用方法

1.数字分级法（NRS）：使用《疼痛程度数字评估量表》（见图 1）对患者疼痛程度进行评估。将疼痛程度用0-10个数字依次表示，0表示无疼痛，10表示能够想象的最剧烈疼痛。交由患者自己选择一个最能代表自身疼痛程度的数字，或由医护人员协助患者理解后选择相应的数字描述疼痛。按照疼痛对应的数字，将疼痛程度分为：轻度疼痛（1-3），中度疼痛（4-6），重度疼痛（7-10）。

**1:**
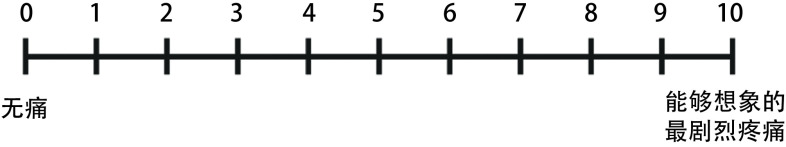
疼痛程度数字评估量表

2.面部表情疼痛评分量表法：由医护人员根据患者疼痛时的面部表情状态，对照《面部表情疼痛评分量表》（见图 2）进行疼痛评估，适用于自己表达困难的患者，如儿童、老年人、存在语言文化差异或其他交流障碍的患者。

**2:**
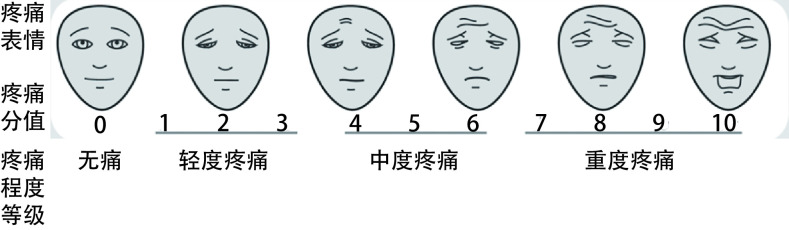
面部表情疼痛评分量表

3.主诉疼痛程度分级法（VRS）：主要是根据患者对疼痛的主诉，可将疼痛程度分为轻度、中度、重度三类。

（1）轻度疼痛：有疼痛，但可忍受，生活正常，睡眠未受到干扰。

（2）中度疼痛：疼痛明显，不能忍受，要求服用镇痛药物，睡眠受到干扰。

（3）重度疼痛：疼痛剧烈，不能忍受，需用镇痛药物，睡眠受到严重干扰，可伴有植物神经功能紊乱或被动体位。

附件2 Karnofsky评分（KPS，百分法）

**Table:** 

体力状况	评分
正常，无症状和体征	100分
能进行正常活动，有轻微症状和体征	90分
勉强进行正常活动，有一些症状或体征	80分
生活能自理，但不能维持正常生活和工作	70分
生活能大部分自理，但偶尔需要别人帮助	60分
常需要人照料	50分
生活不能自理，需要特别照顾和帮助	40分
生活严重不能自理	30分
病重，需要主要和积极的支持治疗	20分
重危，临近死亡	10分
死亡	0分
得分越高，健康状况越好，越能忍受治疗给身体带来的副作用，因而也就有可能接受彻底的治疗，得分越低，健康状况越差，若低于60分，许多有效的抗肿瘤治疗就无法实施。	

附件3 ZPS评分

**Table:** 

0	正常评分
1	症状轻，生活自理，能从事轻体力活动
2	能耐受肿瘤的症状，生活自理，但白天卧床时间不超过50%
3	肿瘤症状严重，白天卧床时间超过50%，但还能起床站立，部分生活自理
4	病重卧床不起
5	死亡

附件4  WHO癌痛三阶梯止痛

轻度疼痛：非甾体类抗炎药（nonsteroidal antiinflammatory drugs, NSAID）±辅助药物

中度疼痛：阿片类止痛药+非甾体类抗炎镇痛药±辅助药物

严重疼痛：强阿片类止痛药+非甾体类抗炎药（NSAID）±辅助药物

附件5  常用癌痛治疗药物表

**Table:** 

分类	药物	药物名称	用法用量
阿片生物碱及其衍生物	可待因	磷酸可待因片	口服，一次15 mg-30 mg，一日30 mg-90 mg；极量：口服，一次100 mg，一日250 mg。
	吗啡	吗啡缓释片	包括硫酸盐或盐酸盐。本品必须整片吞服，不可掰开、碾碎或咀嚼。成人每隔12h按时服用一次，用量应根据疼痛的严重程度、年龄及服用镇痛药史决定用药剂量，个体间可存在较大差异。最初应用本品者，宜从每12h服用10 mg或20 mg开始，根据镇痛效果调整剂量，以及随时增加剂量，达到缓解疼痛的目的。
		吗啡口服即释剂（片剂、口服液）	吗啡片：包括硫酸盐或盐酸盐。口服。成人常用量：一次5 mg-15 mg。一日15 mg-60 mg。对于重度癌痛患者，应按时口服，个体化给药，逐渐增量，以充分缓解癌痛。老年人及肝、肾功能不全患者应酌情减量。硫酸吗啡口服溶液：成人口服常用量为一次5 mg-10 mg，每4h一次，按照拟定的时间表按时服用。可根据患者情况逐渐增量调整，一次用量一般应不超过30 mg，一日用量应不超过0.1 g。根据WHO《癌症疼痛三阶段止痛治疗指导原则》中关于癌症疼痛治疗用药个体化的规定，对癌症患者镇痛使用吗啡应由医师根据病情需要和耐受情况决定剂量。
		吗啡注射剂	盐酸吗啡注射液：皮下注射：成人常用量：一次5 mg-15 mg，一日10 mg-40 mg；静脉注射：成人镇痛时常用量5 mg-10 mg。对于重度癌痛患者，首次剂量范围较大，每日3次-6次，以预防癌痛发生及充分缓解癌痛。硫酸吗啡注射液：可皮下注射。常用量：10 mg-30 mg，每日3次-4次。但患者所需有效剂量及耐受性很不一致，故需逐渐调整使患者不痛为止。一般患者每日用量应不超过100 mg。如长期使用剂量可增高。对身体虚弱或体质量轻于标准的患者，初始剂量应适当减少。
	羟考酮	盐酸羟考酮缓释片	整片吞服，不得掰开、咀嚼或研碎。每12h服用一次，用药剂量取决于患者的疼痛严重程度和既往镇痛药用药史。根据病情仔细滴定剂量，直至理想镇痛。
		羟考酮口服即释剂	盐酸羟考酮胶囊：本品应每隔4 h-6h给药1次，给药剂量应根据患者的疼痛程度和镇痛药的使用既往史而决定。疼痛程度增加，需要增大给药剂量以达到疼痛的缓解。首次服用阿片类药物或用弱阿片类药物不能控制其疼痛的重度疼痛患者，初始给药剂量为5 mg，每隔4 h-6h给药一次。然后应仔细进行剂量滴定，如有必要，可每日一次，以达到疼痛缓解。
	丁丙诺啡	盐酸丁丙诺啡透皮贴剂	每贴使用7 d。初始剂量为最低的丁丙诺啡透皮贴剂剂量（5 *μ*g/h）。应考虑患者先前阿片类药物的用药史，以及患者当前的一般情况和疾病情况。剂量增加应以对补充性止痛药的需求和患者对贴剂的止痛效果的反应为基础。
合成的阿片类药物	芬太尼	芬太尼透皮贴剂	初始剂量应根据患者目前使用阿片类药物剂量而定，建议用于阿片耐受患者。每72h更换一次。
含阿片类药物的复方制剂	对乙酰氨基酚和羟考酮	氨酚羟考酮片	口服给药。成人常规剂量为每6h服用一片，可根据疼痛程度和给药后反应来调整剂量。对于重度疼痛的患者或对阿片类镇痛药产生耐受性的患者，必要时可超过推荐剂量给药。对乙酰氨基酚的用量不宜大于1.5 g/d。
	对乙酰氨基酚和可待因	氨酚待因片	口服：成人，每次1片，每日3次，中度癌症疼痛必要时可由医生决定适当增加。对乙酰氨基酚的用量不宜大于1.5 g/d。
曲马多	曲马多	盐酸曲马多片/胶囊	盐酸曲马多片：口服，一次50 mg-100 mg（1片-2片），必要时可重复给药。日剂量不超过400 mg（8片）。盐酸曲马多胶囊：单次剂量：1个-2个胶囊就少量水服用（50 mg-100 mg盐酸曲马多）。如果镇痛不满意，30 min-60 min以后可再给予1个胶囊。如果疼痛较剧烈，镇痛要求较高，应给予较高的初始剂量（2个胶囊）。每日剂量：一般情况下每日本品总量400 mg（8个胶囊）已足够，但在治疗癌性疼痛和重度术后疼痛时，可使用更高的日剂量。
		盐酸曲马多缓释片	本品应用足量水吞服，不要嚼碎。药片中间有刻痕，可根据剂量需要掰开服用。本品用量视疼痛程度和个人敏感性而定。成人和大于12岁的青少年：通常初始剂量为50 mg-100 mg，每日早晚各一次，如果止痛不满意，剂量可增加到150 mg-200 mg，每日两次。除特殊情况外，每日剂量不应超过400 mg。两次服药的间隔不得少于8 h。

附件6  焦虑、抑郁主要临床表现及治疗

**Table:** 

焦虑	抗焦虑治疗
对癌症复发转移的恐惧和肺部本身的症状有重叠之处 主要症状：心悸或心动过速、呼吸急促、胸痛胸闷、恶心及腹部不适、出汗、发抖或震颤、头晕、感觉异常、潮热、现实感丧失、害怕失控、对死亡的恐惧	轻度焦虑可采用行为干预治疗，如神经肌肉放松训练中度-重度焦虑需要联合应用行为干预和抗焦虑药物
抑郁	抗抑郁治疗
情绪低落或高兴不起来、兴趣缺乏或乐趣丧失、精力体力不足、自信心丧失、自卑、自责、自罪、睡眠障碍、食欲降低、体质量下降等。	轻度抑郁可采用认知-行为治疗和支持性治疗或抗抑郁药物治疗中度-重度抑郁可采用抗抑郁药物治疗联合心理治疗

附件7  肿瘤患者常用抗焦虑药物

**Table:** 

药物	剂量范围	参考
1.苯二氮䓬类		
劳拉西泮	0.25 mg-2.0 mg PO *q4-12h*	无代谢方面不良反应，可用于肝脏肿瘤或转移瘤，减轻恶心和呕吐
阿普唑仑	0.25 mg-1.0 mg PO *q6-24h*	快速起效，快速耐受
奥沙西泮	7.5 mg-15 mg PO *q8-24h*	无代谢方面不良反应
地西泮	2 mg-10 mg PO/IM *q6-24h*	对慢性持续焦虑有效
氯硝西泮	0.5 mg-2.0 mg PO/IM *q6-24h*	对慢性持续焦虑有效，发作性焦虑或有冲动行为
2.抗抑郁药		
帕罗西汀	20 mg-40 mg/d PO	治疗惊恐障碍，镇静作用较强，恶心
艾司西酞普兰	10 mg-20 mg/d PO	治疗惊恐障碍，恶心、疲乏
舍曲林	25 mg-150 mg/d PO	治疗焦虑，恶心
文拉法辛	75 mg-225 mg/d PO	治疗GAD，恶心
曲唑酮	50 mg-100 mg/d PO	治疗伴有抑郁症状的焦虑障碍，头晕、恶心
3.抗精神病药		
奥氮平	2.5 mg-10 mg/d PO	镇静作用较强
喹硫平	25 mg-50 mg/d PO	镇静作用较强

附件8 肿瘤患者常用的抗抑郁药物

**Table:** 

药物	剂量范围	参考	
		临床应用特点	主要不良反应和预警
舍曲林	25 mg/d-150 mg/d	·可改善焦虑、潮热 ·几乎没有药物间相互作用	·胃肠道不良反应如恶心 ·镇静作用较强
氟西汀	10 mg/d-60 mg/d	·可改善潮热 ·半衰期长，停药综合征风险	·可能导致头痛、胃肠功能紊乱、性功能障碍、失眠、坐立不安和减少血小板聚集 ·与低钠血症相关 ·可发生五羟色胺综合征 ·抑制他莫西芬转化为活性代谢物 ·药物间相互作用潜在可能性高
帕罗西汀	20 mg/d-60 mg/d	可用于治疗潮热，但临床不推荐 （原因见不良反应和预警）	·药物间相互作用潜在可能性高（通过CYP450酶发生） ·因半衰期短，停药综合征风险高 ·体质量增加 ·抗胆碱能特性
西酞普兰	20 mg/d-60 mg/d	·可改善焦虑、潮热 ·几乎没有药物间相互作用	·胃肠道不良反应如恶心 ·疲劳 ·QT间期延长的风险
艾司西酞普兰	10 mg/d-20 mg/d	·西酞普兰的S-异构体，作用同西酞普兰 ·副作用较小，几乎没有药物间 ·相互作用	·胃肠道不良反应，如恶心 ·疲劳 ·镇静作用
阿米替林	12.5 mg/d-25 mg/d	·低剂量时可治疗神经病理性疼痛	·抗胆碱能不良反应在肿瘤患者中主要表现为便秘（与阿片类药物会产生交互作用）、口干（粘膜炎风险）以及抗胆碱能毒性（例如：谵妄） ·直立性低血压和心率失常的风险 ·体质量增加和镇静作用
文拉法辛	18.75 mg/d-225 mg/d	·用于治疗神经病理性疼痛和潮热 ·最不可能影响他莫西芬代谢	·在高剂量时升高血压 ·停药综合征风险高 ·恶心、腹泻、失眠、性功能障碍和头痛
度洛西汀	20 mg/d-60 mg/d	用于神经病理性疼痛和潮热	·肝毒性风险，检测肝功能 ·尿储留
米氮平	15 mg/d-45 mg/d	·有助于失眠和刺激食欲 ·止吐作用 ·用于潮热 ·最不可能影响他莫西芬代谢 ·对性功能影响最低 ·有口腔崩解片剂型	·少见粒细胞缺乏症（检测白细胞数量和中性粒细胞绝对值） ·增加脂肪 ·苯丙酮尿症禁用
曲唑酮	25 mg/d-400 mg/d	·作为抗抑郁药使用通常镇静作用太强，适用于非习惯性的催眠 ·抗焦虑作用 ·对性功能影响最小	·罕见不良反应：阴茎持续勃起症 ·避免心肌梗死后使用
安非他酮	75 mg/d-450 mg/d	·对性功能影响最小 ·可用于戒烟 ·不会降低血小板的聚集作用 ·另中枢神经系统兴奋作用	·降低癫痫阈值 ·抑制他莫西芬转化为活性代谢产物 ·失眠和头痛
哌甲酯	5 mg/d-60 mg/d	·快速改善心境 ·提高精神警觉性 ·降低疲劳感	·可引起焦虑和激越 ·可能会降低癫痫发作的阈值 ·高剂量时会导致厌食 ·导致精神病性症状加重或恶化
氟哌噻吨/美利曲辛	1片/d-2片/d	·复方合剂，小剂量用药时具有抗焦虑和抗抑郁作用 ·美利曲辛小剂量应用时具有兴奋作用 ·适用于轻/中度抑郁症，尤其是心因性抑郁、躯体疾病办法抑郁、围绝经期抑郁、酒精依赖及药瘾伴发的抑郁，以及伴发焦虑症的患者 ·耐受性好，不良反应相对较轻	·可能出现轻微口干，大剂量时少见不安或轻微震颤 ·心肌梗死的恢复早期、束支传导阻滞、未经治疗的闭角型青光眼急性酒精、巴比妥类药物及阿片中毒；兴奋或活动过多的患者不宜使用 ·若患者已预先使用了具镇静作用的安定剂，应逐渐减量停用，在与镇静剂同时使用的过程中，应中午以前服用本药（不晚于下午4点），睡前服用镇静药 ·与MAOI之间需有2周的停药间隔 ·停药时应缓慢减药，较少戒断反应
阿戈美拉汀	25 mg/d-50 mg/d	·能在1天-2天内对抑郁相关的睡眠有改善作用 ·停药时不需要逐步递减剂量	·最常见的不良反应为恶心和头晕 ·乙型肝炎或丙型肝炎病毒携带者/患者、肝功能损害患者禁用 ·18岁以下、伴有痴呆的老年患者不应使用、有躁狂或轻症躁狂发作史、伴有中/重度肾功能不全、过量饮酒的患者慎用 ·用药期间应戒酒
